# A TTP-incorporated scoring model for predicting mortality of solid tumor patients with bloodstream infection caused by *Escherichia coli*

**DOI:** 10.1007/s00520-021-06442-z

**Published:** 2021-07-24

**Authors:** Qing Zhang, Hao-Yang Gao, Ding Li, Chang-Sen Bai, Zheng Li, Shan Zheng, Wen-Fang Zhang, Yun-Li Zhou, Si-He Zhang

**Affiliations:** 1 Medical Laboratory Department, National Clinical Research Center for Cancer, Key Laboratory of Cancer Prevention and Therapy, Tianjin’s Clinical Research Center for Cancer, Tianjin Medical University Cancer Institute and Hospital, Tianjin, China; 2 Medical Laboratory Department, Affiliated Hospital of Gansu University of Chinese Medicine, Gansu, China; 3 Department of Cell Biology, School of Medicine, Nankai University, 94 Weijin Road, Nankai District, Tianjin, 300071 People’s Republic of China

**Keywords:** Scoring model, Risk factor, Bacteremia, Time to positivity, Solid tumors

## Abstract

**Background:**

Few mortality-scoring models are available for solid tumor patients who are predisposed to develop *Escherichia coli*–caused bloodstream infection (ECBSI). We aimed to develop a mortality-scoring model by using information from blood culture time to positivity (TTP) and other clinical variables.

**Methods:**

A cohort of solid tumor patients who were admitted to hospital with ECBSI and received empirical antimicrobial therapy was enrolled. Survivors and non-survivors were compared to identify the risk factors of in-hospital mortality. Univariable and multivariable regression analyses were adopted to identify the mortality-associated predictors. Risk scores were assigned by weighting the regression coefficients with corresponding natural logarithm of the odds ratio for each predictor.

**Results:**

Solid tumor patients with ECBSI were distributed in the development and validation groups, respectively. Six mortality-associated predictors were identified and included in the scoring model: acute respiratory distress (ARDS), TTP ≤ 8 h, inappropriate antibiotic therapy, blood transfusion, fever ≥ 39 °C, and metastasis. Prognostic scores were categorized into three groups that predicted mortality: low risk (< 10% mortality, 0–1 points), medium risk (10–20% mortality, 2 points), and high risk (> 20% mortality, ≥ 3 points). The TTP-incorporated scoring model showed excellent discrimination and calibration for both groups, with AUC being 0.833 vs 0.844, respectively, and no significant difference in the Hosmer–Lemeshow test (6.709, *P* = 0.48) and the chi-square test (6.993, *P* = 0.46). Youden index showed the best cutoff value of ≥ 3 with 76.11% sensitivity and 79.29% specificity. TTP-incorporated scoring model had higher AUC than no TTP-incorporated model (0.837 vs 0.817, *P* < 0.01).

**Conclusions:**

Our TTP-incorporated scoring model was associated with improving capability in predicting ECBSI-related mortality. It can be a practical tool for clinicians to identify and manage bacteremic solid tumor patients with high risk of mortality.

**Supplementary Information:**

The online version contains supplementary material available at 10.1007/s00520-021-06442-z.

## Introduction

Advances in surgery combined with targeted or chemotherapies have substantially improved the survival rate of solid tumor patients. However, because of baseline immunodeficiency, cytotoxic treatments, and frequent invasive procedures, solid tumor patients are at high risk of bloodstream infection (BSI). An estimated 5.5–16.4‰ of solid tumor patients developed BSI [1], which pose significant burden on healthcare institutions as well as patients’ families. In light of these considerations, accurate and timely prognostic assessment of the outcome of infected solid tumor patients is essential to triage them for appropriate care and treatment regimens.

*Escherichia coli* (EC) is the most common cause of Gram-negative BSI. The incidence of EC-caused BSI (ECBSI) is increasing in the UK and Europe [2–4]. EC was also the predominant pathogen of BSI in the Asia–Pacific region (26.0% overall) [5]. In China, EC ranked first in the top five bacteremic pathogens from 2011 to 2016 [6]. Multidrug-resistant EC (e.g., extended-spectrum-beta-lactamase, ESBL) has spread in recent decades, and became a major health problem worldwide. This problem is of particular concern among solid tumor patients with immunosuppressed status, who are at high risk of severe sepsis and with BSI-related mortality. Currently, treatment of ECBSI is still a challenge, which makes the morbidity and mortality in infected solid tumor patients high [1, 7].

Several scoring models were established to estimate the BSI-related mortality in infected patients. These scoring models generally adopt risk factors from clinical and laboratory variables. For example, Al-Hasan et al. established a mortality-predictive model by using the Pitt bacteremia score (PBS) together with risk factors including malignancy, liver cirrhosis, and non-urinary/CVC source of BSI [8]. This scoring model is applicable for Gram-negative BSI patients who received adequate empirical antimicrobial therapy. Yishu Tang et al. established a scoring model that is suitable to identify 30-day mortality in hematologically malignant (HM) patients with BSI [9]. Risk factors including in this model are relapsed or uncontrolled malignancy, use of vasopressors, fungemia, acute respiratory failure, and hyperbilirubinemia together with PBS > 3. Other studies about BSI-related mortality-predictive models involve critical patients, sepsis patients, and high-risk patients in the emergency department [10–13]. Obviously, these scoring models were established based on clinical data from relatively homogeneous patients’ population, and could not be applicable to solid tumor patients due to differences in infected pathogen spectrum, patient characteristics, and treatment preference. More importantly, recent studies have shown that blood culture time to positivity (TTP) represents a quantitative surrogate predictor of the severity of BSI. Shorter TTP is associated with poor outcome of patients infected with certain organisms (e.g., *Escherichia coli*, *Staphylococcus aureus*, *Pseudomonas aeruginosa*, and *Candida species*) [14–18]. We hypothesized that TTP may be a useful predictor to solid tumor patients with bacteremia, and may be incorporated into a scoring model for the prediction of mortality. Therefore, the objective of this study was to develop a TTP-incorporated scoring model to identify solid tumor patients at risk of ECBSI-caused mortality.

## Methods

### Setting and study design

This cohort study was conducted in Tianjin Medical University Cancer Institute and Hospital, a 2400-bed medical center providing primary and tertiary care in northern China. The study was evaluated by the Ethics Committee of Tianjin Medical University and deemed exempt from a formal review as no personally identifiable information was collected. The requirement for informed consent from patients was also waived. All patients with blood cultures that yielded *Escherichia coli* between January 2013 and December 2018 were enrolled.

During the study period, the utilization of anti-infection therapies for solid tumor patients was performed according to the guidelines [19, 20]. For patients who had more than one positive culture with the same speciation and sensitivity, only the first one was counted. Clinical data on demographic characteristics; comorbid condition; and treatment course during hospitalization, discharge diagnosis, and outpatient follow-up were collected through electronic medical record review. Exclusion criteria for patients were (1) blood cultures’ contamination; (2) polymicrobial BSI (blood culture samples which showed different bacterial strains within 48 h); (3) a recurrent BSI occurring in the same patient; (4) incomplete clinical data including loss to follow-up; and (5) < 18 years old.

Solid tumor patients with ECBSI were assigned to the development group (two-thirds of the total number) and the validation group (one-third) with random distribution function of SPSS software. The primary outcome was in-hospital mortality after the onset of ECBSI. The survivors and non-survivors were compared to identify the potential risk factors for mortality. The development group was used to create mortality-scoring model, and scoring performance of the model was assessed by the validation group.

### Microbiology analyses

Blood cultures were obtained by inoculation of blood samples into aerobic and anaerobic flasks. Blood samples were incubated in a BACTEC FX400 Automated Blood Culture System (Becton Dickinson, USA) in the clinical microbiology laboratory, and monitored for CO_2_ production every 10 min for 5 days. The time from blood culture inoculation to the detection of a positive signal was reported as TTP [14]. In patients with multiple sets of positive blood culture, the shortest TTP was used for analysis. All culture-positive blood samples were subcultured to identify bacterial isolates, according to the VITEK 2 automated microbiology system (bioMérieux, France) with conventional biochemical methods. All antibiotic susceptibilities were evaluated via minimal inhibitory concentration (MIC) according to the Clinical Laboratory Standard Institute (CLSI) criteria [21]. ESBL-EC was further confirmed by performing the double-disc synergy tests [22]. *Escherichia coli* ATCC25922 (negative control) and *Klebsiella pneumoniae* ATCC 700,603 (positive ESBL producer) were used as quality controls.

### Variables and definitions

The following variables of each patient were collected at the onset of ECBSI: malignancy diagnosis, disease status, comorbidities, and antibiotic therapy. As previously described, ECBSI was defined as the isolation of *Escherichia coli* from at least one bottle of blood culture specimens from patients with compatible clinical signs or symptoms [1]. The date of the first positive blood culture was regarded as the onset date of BSI. Disease status was assessed by the most recently available tumor biopsy and categorized as remission, relapsed, and uncontrolled malignancy, following the previous definition [19]. Neutropenia was defined as an absolute neutrophil count (ANC) of < 500 cells/mm^3^ [23]. Acute respiratory distress syndrome (ARDS) was defined as previously described [23]. Inappropriate antibiotic therapy was defined as the empiric administration of antimicrobial agents that were ineffective against the causative microorganism either in vivo or in vitro [24]. In-hospital mortality was defined as death by any cause within the first 30 days after the onset of BSI during hospitalization [8].

### Statistical analyses and model setup

Median values and interquartile range (IQR) were calculated for continuous variables, and percentages were used for categorical variables. The Mann–Whitney *U* test was used to compare continuous variables, and the categorical variables were analyzed with chi-square test. The cutoff values were set according to clinical practice or laboratory references. Potential risk factors for in-hospital mortality with *P* < 0.05 on univariable regression analysis were further investigated by multivariable regression analysis, using a backward selection method. Significant mortality-associated risk factors were assigned weighted points that were proportional to their β regression coefficient values. The risk scores were calculated for each patient. Patients were categorized at deciles of risk score, and then divided into three catalogues: low risk (< 10% predicted mortality), medium risk (10–20% predicted mortality), and high risk (> 20% predicted mortality). The predictive mortality was calculated for each risk catalogue, and the discriminatory ability of the scoring model was assessed by the area under the receiver operating characteristic (ROC) curve (AUC). Following the model establishment, validation was performed by using variables from the validation group. The difference of AUC between the two groups was compared using the chi-square test. The positive predictive value (PPV), negative predictive value (NPV), predictive sensitivity, and specificity were calculated at different cutoff values. The best cutoff value for mortality-risk stratification was determined based on Youden index statistics. Data were analyzed with SPSS software. All *P* values are two-sided, and a *P*-value < 0.05 was considered statistically significant.

## Results

Clinical data of 535 solid tumor patients with ECBSI were collected. Of those, 23 recurrent episodes and three incomplete data of cases were excluded from the analysis. Thus, a total of 509 solid tumor patients with a first episode of ECBSI were finally enrolled. Demographic and clinical characteristics of solid tumor patients are shown (Table 1). Median age was 61 years and 30-day in-hospital mortality was 22.2% for overall patients. There was no significant difference between the two groups in all the characteristics under investigation (Table 1), indicating the grouping was random and even.

**Table 1:** Demographic and clinical characteristics of solid tumor patients with ECBSI

Variables	Development group *n* (%) (*n* = 315)	Validation group *n* (%) (*n* = 194)	*P* value
Gender			
Male	159 (50.5%)	98 (50.5%)	0.993
Female	156 (49.5%)	96 (49.5%)	
Age in years, median (IQR)	61 (53–68)	61 (54–68)	0.788
Fever ≥ 39 °C	199 (63.2%)	129 (66.5%)	0.428
Comorbidities			
Hypertension	89 (28.3%)	55 (28.4%)	0.984
Chronic heart disease	41 (13.0%)	23 (11.9%)	0.682
Diabetes mellitus	39 (12.4%)	21 (10.8%)	0.580
Underlying tumor			
Lung tumor	27 (8.6%)	14 (7.2%)	0.420
Hepatocellular tumor	67 (21.3%)	38 (19.6%)	
Gastroenterological tumor	37 (11.7%)	34 (17.5%)	
Mammary tumor	18 (5.7%)	13 (6.7%)	
Gynecological tumor	35 (11.1%)	21 (10.8%)	
Pancreatic tumor	60 (19.0%)	33 (17.0%)	
Colorectal tumor	35 (11.1%)	15 (7.7%)	
Genitourinary tumor	16 (5.1%)	18 (9.3%)	
Others^a^	20 (6.3%)	8 (4.1%)	
ESBL	156 (49.5%)	105 (54.1%)	0.313
ICU admission	29 (9.2%)	14 (7.2%)	0.433
Inappropriate antibiotic therapy, *n* (%)	87 (27.6)	47 (24.2%)	0.384
Metastasis	163 (51.7%)	91 (46.9%)	0.282
Site of infection acquisition, *n* (%)			
Biliary infection	99 (31.4%)	59 (30.4%)	0.107
Pulmonary infection	44 (17%)	17 (8.8%)	
Abdominal infection	69 (21.9%)	60 (30.9%)	
Urinary tract infection	42 (13.3)	26 (13.4%)	
Catheter-related	13 (4.1%)	4 (2.1%)	
Others	24 (7.6%)	9 (4.6%)	
Unknown origin	24 (7.6%)	19 (9.8%)	
ARDS at admission	15 (4.8%)	8 (4.1%)	0.729
Blood transfusion	99 (31.4%)	57 (29.4%)	0.604
Central line	138 (43.8%)	82 (42.3%)	0.688
TTP ≤ 8 h	63 (20%)	42 (21.6%)	0.655
Neutropenia	51 (16.2%)	24 (12.4%)	0.232
30-day mortality	73 (23.2%)	40 (20.6%)	0.500

^a^Meningioma, 7; metastatic encephalon, 3; bone and soft tissue tumor, 13; thyroid neck tumor, 5

Variables from the development group were used to establish a risk-scoring model for predicting the mortality. Results from univariable regression analysis showed that fever ≥ 39 °C, inappropriate antibiotic therapy, metastasis, central line, blood transfusion, hypertension, ARDS at admission, and TTP ≤ 8 h were potential risk factors related to the mortality (Table 2). The multivariable regression model, which incorporates all potential risk factors from the univariable regression analysis, identified six predominant predictors for in-hospital mortality: fever ≥ 39 °C (OR = 2.93, 95% CI = 1.388–6.185, *p* = 0.005), inappropriate antibiotic therapy (OR = 3.636, 95% CI = 1.895–6.975, *p* < 0.001), metastasis (OR = 2.972, 95% CI = 1.443–6.119, *p* = 0.003), ARDS (OR = 10.159, 95% CI = 2.678–38.529, *p* = 0.001), blood transfusion (OR = 2.884, 95% CI = 1.511–5.505, *p* = 0.001), and TTP ≤ 8 h (OR = 2.64, 95% CI = 1.28–5.444, *p* = 0.009) (Table 2). These six independent predictors were then combined to further calculate the risk score of mortality in solid tumor patients with ECBSI.

**Table 2:** Regression analyses of mortality-associated predictors in solid tumor patients with ECBSI

Risk factors	Survivors *n* (%) (*n* = 242)	Non-survivors *n* (%) (*n* = 73)	*P* value	OR (95% CI)	*P* value
Gender					
Male	120 (49.6%)	39 (53.4%)	0.565		
Female	122 (50.4%)	34 (46.6%)			
Age in years, median (IQR)	62 (54–68)	60 (52–70)	0.925		
Fever ≥ 39 °C	140 (57.9%)	59 (80.8%)	** < 0.001**	2.93 (1.388–6.185)	**0.005**
ESBL	119 (49.2%)	37 (50.7%)	0.821		
ICU admission	20 (8.3%)	9 (12.3%)	0.292		
Comorbidities					
Hypertension	75 (31.0%)	14 (19.2%)	**0.045**	0.66 (0.302–1.445)	0.229
Chronic heart disease	31 (12.8%)	10 (13.7%)	0.862		
Diabetes mellitus	27 (11.2%)	12 (16.4%)	0.240		
Inappropriate antibiotic therapy, *n* (%)	48 (19.8%)	39 (53.4%)	** < 0.001**	3.636 (1.895–6.975)	** < 0.001**
Metastasis	105 (43.4%)	58 (79.5%)	** < 0.001**	2.972 (1.443–6.119)	**0.003**
Site of infection acquisition, *n* (%)					
Biliary infection	79 (32.6%)	20 (27.4%)	0.368		
Pulmonary infection	37 (15.3%)	7 (9.6%)			
Abdominal infection	50 (20.7%)	19 (26%)			
Urinary tract infection	32 (13.2%)	10 (13.7%)			
Catheter-related	7 (2.9%)	6 (8.2%)			
Others	18 (7.4%)	6 (8.2%)			
Unknown origin	19 (7.9%)	5 (6.8%)			
ARDS at admission	4 (1.7%)	11 (15.1%)	** < 0.001**	10.159 (2.678–38.529)	**0.001**
Blood transfusion	63 (26.0%)	36 (49.3%)	** < 0.001**	2.884 (1.511–5.505)	**0.001**
Central line	116 (47.9%)	22 (30.1%)	**0.006**	0.523 (0.257–1.061)	0.072
TTP ≤ 8 h	38 (15.7%)	25 (34.2%)	**0.001**	2.64 (1.28–5.444)	**0.009**
Neutropenia	35 (14.5%)	16 (21.9%)	0.133		

**Fig. 1:**
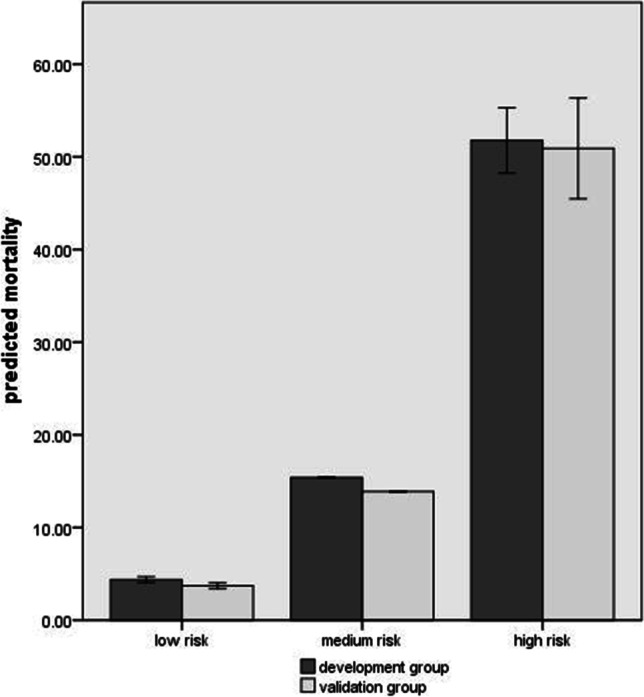
Predicted mortality (95% CI) of TTP-incorporated model in development group versus validation group

**Fig. 2:**
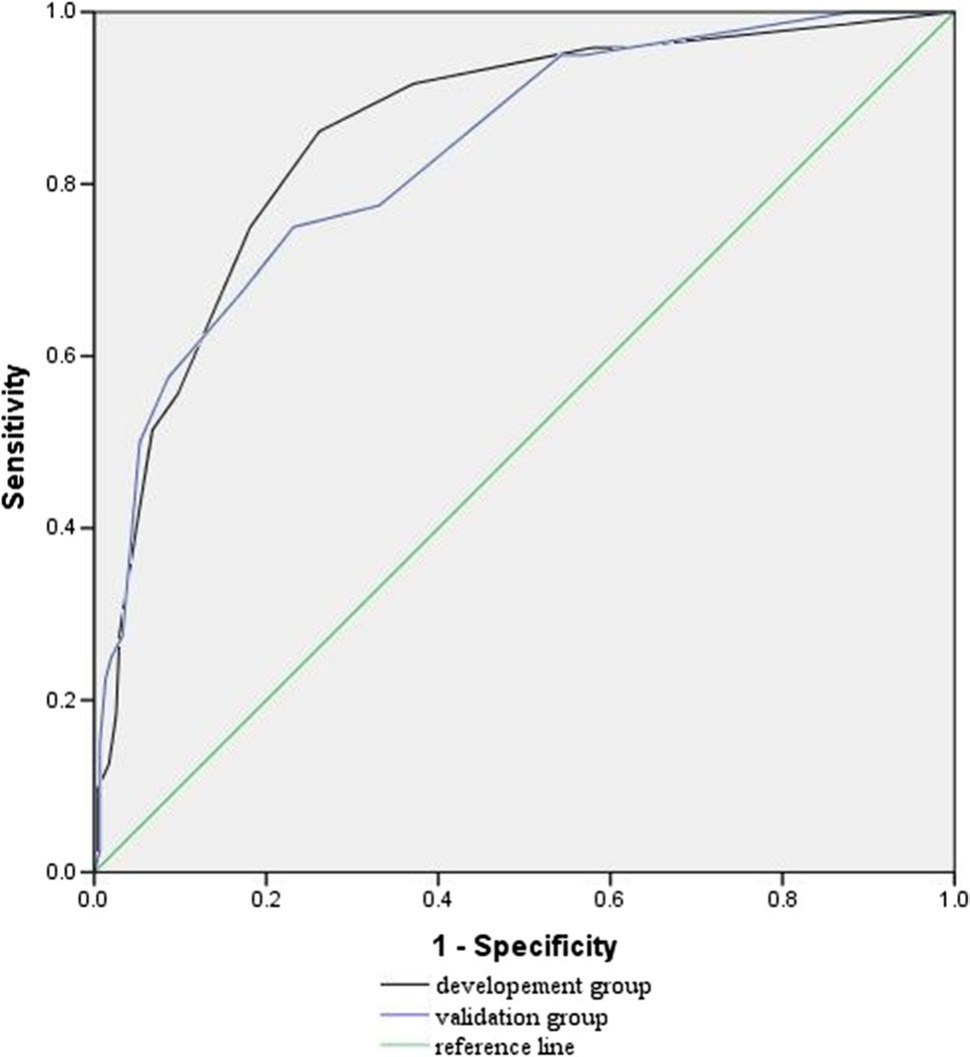
Comparison of the area under the receiver operating characteristic (ROC) curve between development and validation groups

Point values assigned to each predictor within the established TTP-incorporated and the no TTP-incorporated (NTTP) scoring models are shown (Table 3). In development group, the predicted mortality for low-risk, medium-risk, and high-risk categories by TTP-incorporated model was 4.38%, 15.39%, and 51.77%, respectively, while those for validation group were 3.72%, 13.88%, and 50.09%, respectively (Fig. 1). No difference for the predicted mortality was observed between the two groups. However, this prediction by two models was distinct in both groups with a proportion of patients in low-risk, medium-risk, and high-risk groups of 4.12%, 14.8%, and 51.46% (for TTP model), and 7.83%, 17.97%, and 44.01% (for NTTP model), respectively (Fig. S1). The TTP model showed excellent discrimination, with AUC being 0.833 (95% CI = 0.779–0.887) in development group versus 0.844 (95% CI = 0.774–0.913) in validation group (Fig. 2). Similar discrimination in two groups was found with AUC being 0.837 (95%CI = 0.795–0.880) by TTP model versus 0.817 (95%CI = 0.774–0.861) by NTTP model (Fig. S2). The model also had good calibration in both groups, without difference after the Hosmer–Lemeshow test (6.709, *P* = 0.48) and the chi-square test (6.993, *P* = 0.46). In both development and validation groups, our model showed reliable and consistent increase in mortality with increasing score (Fig. S3). Predictive sensitivity and specificity, PPV and NPV at different thresholds of the two scoring models are shown (Table 4). The Youden index indicated that the TTP-incorporated scoring model performed best at a cutoff value of ≥ 3 points (Table 4, Table S1), whereas the NTTP scoring model performed best at a cutoff value of ≥ 2 points (Table 4). Prediction sensitivity and NPV increased along with the decrease of cutoff values, indicating our scoring model provides highly diagnostic accuracy.

**Table 3:** Risk-scoring model of in-hospital mortality for solid tumor patients with ECBSI

Predictors	*β* coefficient	OR (95% CI)	*P* value	Score points
TTP-incorporated model (TTP)				
TTP ≤ 8 h	1.025	2.788 (1.360–5.716)	**0.005**	1
Inappropriate antibiotic therapy	1.299	3.667 (1.921–7.000)	** < 0.001**	1
ARDS at admission	2.172	8.777 (2.337–3.296)	**0.001**	2
Blood transfusion	1.047	2.849 (1.499–5.414)	**0.001**	1
Metastasis	1.219	3.385 (1.67–6.861)	**0.001**	1
Fever ≥ 39 °C	1.110	3.034 (1.448–6.357)	**0.003**	1
No TTP-incorporated model (NTTP)				
Inappropriate antibiotic therapy	1.279	3.59 (1.91–6.76)	**0.000**	1
ARDS at admission	2.044	7.72 (2.09–28.48)	**0.002**	1
Blood transfusion	1.042	2.84 (1.51–5.33)	**0.001**	1
Metastasis	1.404	4.07 (2.04–8.11)	**0.000**	1
Fever ≥ 39 °C	1.098	3 (1.44–6.23)	**0.003**	1

**Table 4:** Accuracy of the risk score in the identification of ECBSI-associated mortality among solid tumor patients, stratified according to the cutoff value of the risk score

Cutoff	Sensitivity, % (95% CI)	Specificity, % (95% CI)	PPV, % (95% CI)	NPV, % (95% CI)	Case number (% of entire cohort)	Youden index
TTP model						
≥ 1	99.12 (94.45–99.95)	13.38 (10.27–17.23)	24.62 (20.78–28.89)	98.15 (88.82–99.9)	455 (89.39)	0.128
≥ 2	92.92 (86.1–96.67)	45.96 (40.99–51.01)	32.92 (27.84–38.41)	95.79 (91.58–98.03)	319 (63.49)	0.398
≥ 3	76.11 (66.99–83.41)	79.29 (74.9–83.11)	51.19 (43.4–58.93)	92.08 (88.56–94.62)	168 (33.01)	0.568
≥ 4	44.25 (35.01–53.88)	92.42 (89.25–94.75)	62.5 (50.92–72.87)	85.31 (81.53–88.46)	80 (15.72)	0.381
≥ 5	15.04 (9.26–23.28)	97.47 (95.25–98.71)	62.96 (42.47–79.92)	80.08 (76.18–83.5)	27 (5.3)	0.138
≥ 6	5.31 (2.18–11.67)	97.47 (95.25–98.71)	37.5 (16.28–64.13)	78.3 (74.34–81.8)	16 (3.14)	0.039
NTTP model						
≥ 1	98.23 (93.12–99.69)	24.49 (20.4–29.09)	27.07 (22.88–31.7)	97.98 (92.19–99.65)	410 (80.55%)	0.232
≥ 2	82.3 (73.75–88.6)	70.2 (65.39–74.6)	44.08 (37.31–51.06)	93.29 (89.66–95.75)	211 (41.45%)	0.538
≥ 3	40.71 (31.69–50.37)	91.92 (88.67–94.32)	58.97 (47.25–69.8)	84.45 (80.61–87.67)	78 (15.32%)	0.340
≥ 4	13.27 (7.87–21.26)	96.46 (94–97.98)	51.72 (32.9–70.11)	79.58 (75.64–83.04)	29 (5.7%)	0.110
= 5	4.42 (1.64–10.53)	97.98 (95.9–99.06)	38.46 (15.13–67.72)	78.23 (74.28–81.73)	13 (2.55%)	0.036

## Discussion

In this study, we developed a new TTP-incorporated scoring model to predict the bacteremia-associated mortality, and evaluated its capability of risk stratification among solid tumor patients. To our knowledge, this is the first mortality-scoring model established for solid tumor patients with ECBSI. Although one study reported a TTP-incorporated scoring model [25], it was designed to stratify the risk of nontyphoid Salmonella-caused vascular infection (NTSVI). As nontyphoid Salmonella bacteremia is not frequent, the TTP-NTSVI scoring model is not applicable to bacteremic solid tumor patients.

The requirement to stratify solid tumor patients at risk of in-hospital mortality is particularly relevant to those with bloodstream infections. Our established scoring model utilizing TTP and reliable predictors based on modern epidemiology was associated with improved mortality-prediction performance. This performance was well-evidenced both in the development and validation groups, as the prediction sensitivity of our scoring model was improved with the decrease of cutoff values (Table 4). Although at a cutoff value of 3 the prediction sensitivity for both groups was relatively low (76.71% and 75%, respectively), high prediction specificity improved the targeting of solid tumor patients with high mortality risk. Our model showed the predicted mortality increased along with increasing score (Fig. S3). This indicates that solid tumor patients enrolled in this study were likely to have a mild infection and a low mortality at a scale of 0 to 1. If the score increased, the patients might have a severe infection and a high risk of mortality. Therefore, our new TTP-incorporated scoring model could be used as a screening tool to quickly identify high-risk solid tumor patients who may benefit from early intervention.

TTP is a newly developed laboratory indicator. Based on the assessment of initial bacterial inoculum in cultured blood, it was proved to be a useful predictor for the severity of bacteremia, and associated with clinical outcomes of patients with *S. aureus*, *Streptococcus pneumoniae*, *Escherichia coli*, *Klebsiella pneumoniae*, and *Pseudomonas aeruginosa* bacteremia [14–18, 26]. However, prior to this study, the association of shorter TTP with higher risk of mortality was mainly reported in non-tumor patients with *Klebsiella pneumoniae* bacteremia [26]. In this study, we showed that TTP was a dominant predictor for in-hospital mortality in solid tumor patients with ECBSI (Table 2). Our study therefore supports the idea that TTP is a power to provide both prognostic and diagnostic information for physicians in treating solid tumor patients with bacteremia.

In spite of the updated guidelines for the management of bacteremic patients, in-hospital mortality due to BSI in cancer patients remains high. We found in this study that inappropriate antibiotic therapy was associated with high mortality of solid tumor patients with ECBSI (Table 2). Reducing mortality by the rapid initiation of empirical antibiotic therapy for cancer patients is undisputed. However, the frequent cases in the clinic are in vitro susceptibility tests were sensitive, whereas the antibiotic application in solid tumor patients with ECBSI was ineffective. This implies that there might have certain pathophysiological variables in infected solid tumor patients, especially those in advanced stages. Therefore, it is necessary to consider the optimal dosing and tailored individual regimens of antimicrobials for tumor patients with ECBSI.

ARDS is more often fatal in infected patients [27]. In this study, the mortality of bacteremic solid tumor patients with ARDS was accounted for 15.1% (Table 2). This may be due to solid tumor patients undergoing extensive tumor resections, in particular involving the respiratory and gastrointestinal tracts, who are at greater risk of developing postoperative nosocomial infections. In addition, they may have severe acute lung injury related to chemotherapeutic agents and radiation [1]. Given the high mortality associated with ARDS, efforts should be taken to prevent ARDS in solid tumor patients with ECBSI. Early utilization of high-resolution chest CT-scan, serological tests such as galactomannan antigen and bronchoscopy with bronchodilator lavage, could reduce the in-hospital mortality of solid tumor patients with ARDS.

It is a remarkable fact that the blood transfusion as a mortality predictor in bacteremic solid tumor patients has not been reported (Table 2 and Table 3). Anemia is frequently observed in cancer patients, either due to chronic illness or active bleeding. Blood transfusion supplements blood volume and improves microcirculation, but the increased plasma protein may promote coagulation. One study reported transfusion of blood components actually led to worse outcomes in tumor patients [1]. Thus, blood transfusion should be regarded as personalized medicine, carefully considering the bacteremic status of solid tumor patient in order to reduce the in-hospital mortality.

There are two limitations in this study. First, since the current study was performed at a single center in China, liver cancer comprises the most common solid tumor, followed by pancreatic cancer. Liver cancer is less common in the North America and Europe. The difference in expected tumor type would impact the predictive power of this model outside of China. Second, no uniform standard is available for variables in the predictive model and it needs further validation by multiple center research in the future.

## Conclusions

Unlike the HM patients with bacteremia, among those ICU admission, ESBL and neutropenia are the frequent predictors of ECBSI-caused mortality [9, 24]. In bacteremic solid tumor patients, TTP ≤ 8 h, inappropriate antibiotic therapy, ARDS at admission, blood transfusion, fever ≥ 39 °C, and metastasis are predominant predictors associated with ECBSI-caused mortality. We developed a mortality-risk-scoring model that incorporates TTP with other five reliable predictors in solid tumor patients with ECBSI. Our TTP-incorporated scoring model improved the mortality-predictive capability in solid tumor patients with bacteremia. It can be a practical tool for clinicians to identify and manage bacteremic solid tumor patients with high risk of mortality.

## Supplementary Information

Below is the link to the electronic supplementary material.

Supplementary file1 (DOCX 57 KB)

## Data Availability

The data of this study are available from the corresponding author upon request.

## Code availability

N/A.

## Abbreviations

TTP: Time to positivity
EC: *Escherichia coli*
BSI: Bloodstream infection
ECBSI: *Escherichia coli*–Caused bloodstream infection
ARDS: Acute respiratory distress
IQR: Interquartile range
ROC: Receiver operating characteristic
AUC: Area under a ROC curve
PPV: Positive predictive value
NPV: Negative predictive values
HM: Hematological malignant
MIC: Minimum inhibitory concentration
CLSI: Clinical and Laboratory Standards Institute
