# Supplementary material for: A TTP-incorporated scoring model for predicting mortality of solid tumor patients with bloodstream infection caused by Escherichia coli
Source: Support Care Cancer. 2021 Jul 24;30(1):413–21. doi: 10.1007/s00520-021-06442-z (PMC8636427; doi:10.1007/s00520-021-06442-z)
Supplement: Supplementary file 1 — Supplementary file1 (DOCX 57 KB) [file 520_2021_6442_MOESM1_ESM.docx]

**Table S1** Risk-scoring performance of the TTP-incorporated mortality-prediction model

| Cut-off | Sensitivity,  % (95% CI) | Specificity,  % (95% CI) | PPV,  % (95% CI) | NPV,  % (95% CI) | | Case number  (% of entire cohort) | Youden index |
| --- | --- | --- | --- | --- | --- | --- | --- |
| Development group | | | | | | | |
| ≥1 | 98.63(91.57-99.93) | 14.05(10.05-19.22) | 25.71(20.78-31.33) | | 97.14(83.38-99.85) | 280(88.89) | 0.130 |
| ≥2 | 93.15(84.07-97.45) | 45.45(39.1-51.96) | 34(27.56-41.07) | | 95.65(89.65-98.39) | 200(63.49) | 0.395 |
| ≥3 | 76.71(65.09-85.48) | 78.51(72.69-83.41) | 51.85(42.08-61.49) | | 91.79(86.96-95) | 108(34.29) | 0.566 |
| ≥4 | 45.21(33.68-57.24) | 91.32(86.86-94.42) | 61.11(46.88-73.77) | | 84.67(79.59-88.71) | 26(8.25) | 0.377 |
| ≥5 | 13.7(7.12-24.21) | 97.52(94.42-98.99) | 62.5(35.87-83.72) | | 78.93(73.78-83.32) | 11(3.49) | 0.121 |
| ≥6 | 5.48(1.77-14.16) | 97.52(94.42-98.99) | 40(13.69-72.63) | | 77.38(72.18-81.87) | 6(1.9) | 0.037 |
| Validation group | | | | | | | |
| ≥1 | 100(89.09-100) | 12.34(7.78-18.84) | 22.86(17.01-29.93) | 100(79.08-100) | | 175(55.56) | 0.126 |
| ≥2 | 46.75(38.73-54.94) | 31.09(23.1-40.33) | 96(87.97-98.96) | 75(58.48-86.75) | | 119(37.78) | 0.402 |
| ≥3 | 75(58.48-86.75) | 80.52(73.2-86.28) | 50(36.95-63.05) | 92.54(86.35-96.16) | | 60(19.05) | 0.571 |
| ≥4 | 42.5(27.42-58.99) | 94.16(88.86-97.12) | 65.38(44.37-82.06) | 86.31(79.96-90.95) | | 54(17.14) | 0.385 |
| ≥5 | 17.5(7.89-33.36) | 97.4(93.07-99.16) | 63.64(31.61-87.63) | 81.97(75.46-87.1) | | 16(5.08) | 0.168 |
| ≥6 | 5(0.87-18.21) | 97.4(93.07-99.16) | 33.33(6-75.89) | 79.79(73.19-85.14) | | 10(3.17) | 0.043 |


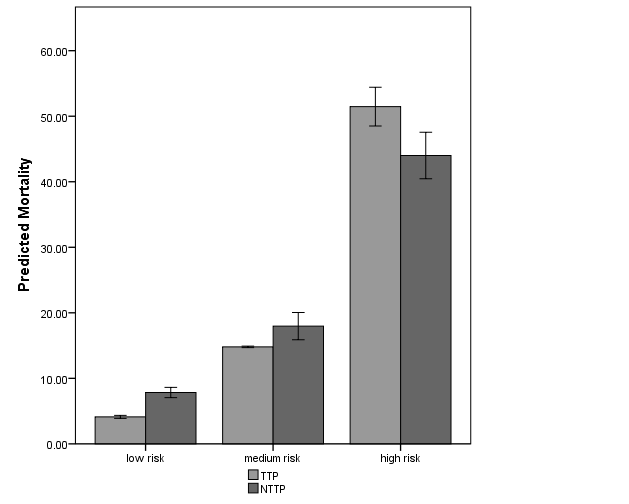


**Fig. S1** Predicted mortality (95% CI) of the mortality-scoring models

Note: TTP: no TTP-incorporated model, NTTP: no TTP-incorporated model


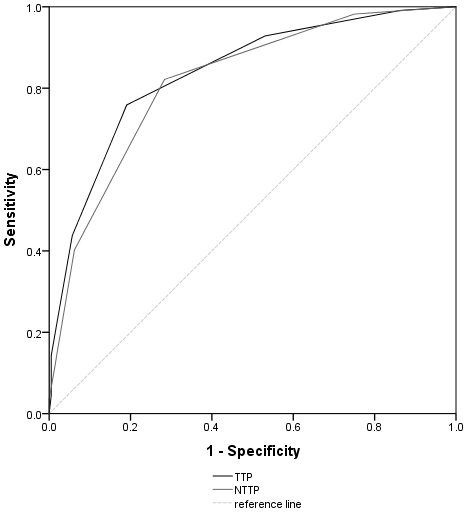


**Fig. S2** Receiver operating characteristic (ROC) curves of the mortality-scoring models

Note: TTP: no TTP-incorporated model, NTTP: no TTP-incorporated model


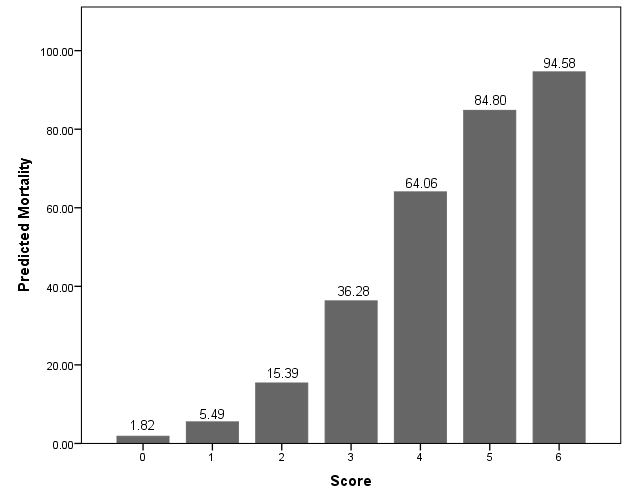


**Fig. S3** Distribution of scores by the TTP-incorporated model
